# Current advances in the development of SARS-CoV-2 vaccines

**DOI:** 10.7150/ijbs.52569

**Published:** 2021-01-01

**Authors:** Annoor Awadasseid, Yanling Wu, Yoshimasa Tanaka, Wen Zhang

**Affiliations:** 1 Lab of Chemical Biology and Molecular Drug Design, College of Pharmaceutical Science, Zhejiang University of Technology, Hangzhou, 310014, China.; 2 Lab of Molecular Immunology, Virus Inspection Department, Zhejiang Provincial Center for Disease Control and Prevention, Hangzhou, 310051, China.; 3 Department of Biochemistry & Food Sciences, University of Kordofan, El-Obeid, 51111, Sudan.; 4 Center for Medical Innovation, Nagasaki University, 1-7-1 Sakamoto, Nagasaki 852-8588, Japan.

**Keywords:** SARS-CoV-2, COVID-19, coronavirus, vaccines, clinical trial

## Abstract

Coronavirus disease 2019 (COVID-19) caused by severe acute respiratory syndrome coronavirus 2 (SARS-CoV-2) is now a global pandemic that has wreaked havoc globally, which has put a heavy toll on public health, lives, and the world economy. Vaccination is considered as one of the greatest successes in medical history. Based on prior experience with the development of SARS-CoV vaccines, all COVID-19 vaccines must be subjected to the tests for protective effects and harmful risks derived from antibody-dependent enhancement that may contribute to augmented infectivity and/or eosinophilic infiltration. The SARS-CoV-2 vaccine is now being developed urgently in several different ways. China is regarded as one of the world's leading countries in SARS-CoV-2 vaccine development, up to date the last inactivated vaccine international clinical (Phase III) trial was launched in the United Arab Emirates by Sinopharm China National Biotec Group (CNBG). In this review, we outline the current status of vaccine development against clinically relevant SARS-CoV-2 strains, anticipating that such attempts would help create efficacious and sage SARS-CoV-2 vaccines.

## Introduction

A cluster of pneumonia patients emerged in Wuhan, Hubei province, China, in December 2019 1. The etiologic agent was quickly recognized as a previously unreported β-coronavirus. The World Health Organization (WHO) named the virus severe acute respiratory syndrome coronavirus 2 (SARS-CoV-2) and the disease it causes coronavirus disease 2019 (COVID-19) 2, 3. As of 23 October 2020, SARS-CoV-2 has affected more than 9,678,494 people worldwide, contributing to more than 1,143,357 deaths, with a mortality rate of 2.72% 4. The world began witnessing the first of a string of fatal coronavirus outbreaks of 2002-2003. The disease was named severe acute respiratory syndrome (SARS), and the etiological agent was SARS coronavirus (SARS-CoV), in which high fever was the initial symptom of the disease, and gradually shifting to shortness of breath and pneumonia 5. SARS evolved in the southern part of China and spread to other countries, reaching 8096 cases and culminating in 774 deaths in 26 countries 6. No vaccine had become commercially available despite efforts in the scientific world, and no case of SARS has been reported since 2004 5. Then, another coronavirus disease called Middle East respiratory syndrome (MERS) appeared in September 2012. Whereas mild respiratory symptoms usually characterized symptoms of infectious diseases observed in Saudi Arabia, symptoms of MERS often progressed into acute respiratory distress syndrome (ARDS) and death 7. There were 27 countries afflicted by the outbreak, resulting in 2494 cases and 858 deaths 8. Although MERS cases have been still recorded since 2015, there was no significant data to announce it as an epidemic 9.

**Figure 1:**
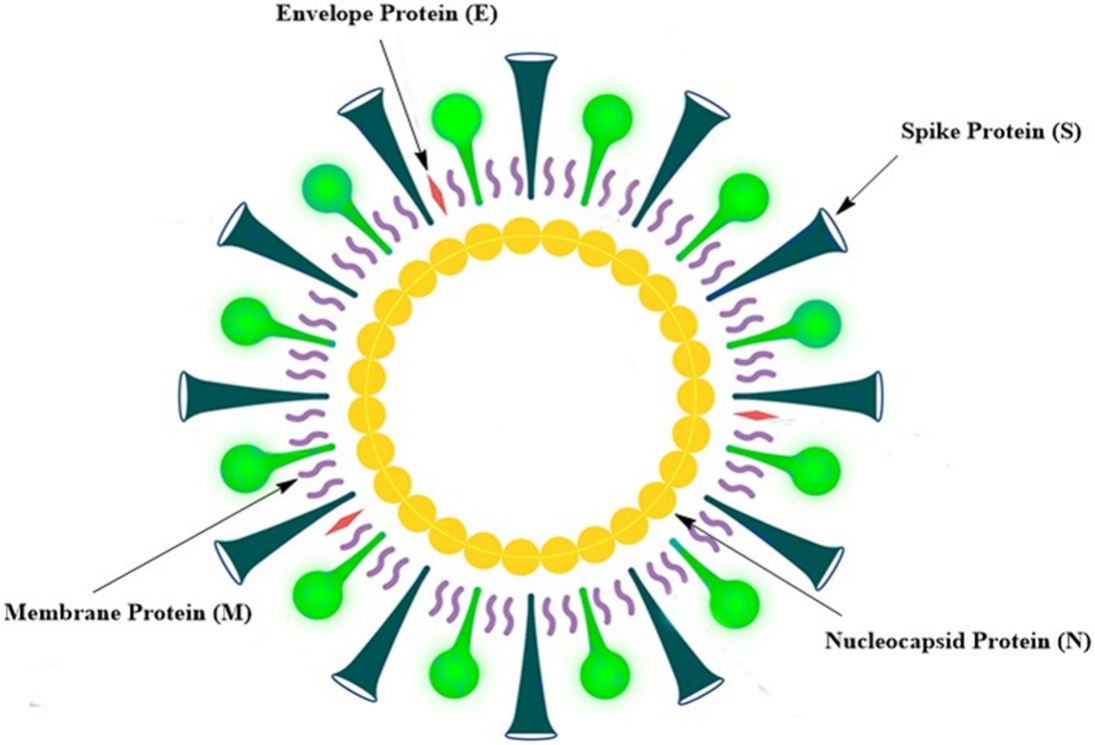
Schematic representation of the SARS-CoV-2 structure.

No approved vaccine is available for MERS, as is the case for SARS. There are various reasons for the failure in the development of SARS and MERS vaccines. In the case of MERS, the vaccine development was hampered in pre-clinical stages due to the lack of viable and cost-effective small animal models 10. In addition, little attention has been paid to the development of MERS vaccines, because MERS incidence has been sporadic and geographically restricted 10. When it comes to SARS, it is difficult to invest in the development of SARS vaccines, because the incidence of SARS has not been reported since 2014, suggesting that SARS-CoV had disappeared 10. Symptoms of COVID-19 caused by SARS-CoV-2 are mostly mild, such as fever, coughing, and breathlessness. In older adults and those with chronic diseases; however, many severe symptoms could occur, including severe pneumonia and organ dysfunction 2, 11. Although no vaccines are currently available for SARS and MERS, previous and ongoing attempts to produce vaccines against such diseases may be of considerable advantage for creating an effective SARS-CoV-2 vaccine 10. SARS-CoV-2 is a positive-sense single-stranded RNA virus. The SARS-CoV-2 genome is about 29,700 nucleotides long and has a 79.5% sequence similarity with SARS-CoV; it has a 5ʹ end long ORF1ab polyprotein that encodes 15 or 16 non-structural proteins 12. The 3ʹ end genome encodes four main structural proteins, such as spike (S) protein, nucleocapsid (N) protein, membrane (M) protein, and protein envelope (E) (**Fig. 1**) 13. SARS-CoV-2 binds to angiotensin receptor conversion enzyme 2 (ACE2) expressed on host cells for viral entry and eventual pathogenesis 14. Vaccines are the best reliable and cost-effective way to avoid and manage infectious diseases 15. The COVID-19 pandemic and the resulting increase in deaths worldwide have rendered the development of an effective SARS-CoV-2 vaccine urgently important.

## SARS and MERS coronavirus vaccines

Following the 2002-2003 SARS outbreak, many laboratories worldwide set out to develop vaccines for the disease 10. Most of the subunit vaccines have been developed based on the viral S glycoprotein 10, which was used for the viral entry 16. Consequently, the immunization with the vaccine might elicit robust immune responses to this protein and exhibit major impacts on preventing viral entry into host cells during natural infections. All possible vaccine types were tested for pre-clinical trials, including live-attenuated virus vaccines, inactivated virus vaccines, recombinant viral vector vaccines, DNA vaccines, mRNA vaccines, virus-like particle vaccines (VLPs), and subunit vaccines 10. The whole SARS-CoV was used for the development of live-attenuated and inactivated virus vaccines 10. The virus was made non-replicative either by removing components of the virus genome or by utilizing physical or chemical processes, by which the infectivity was significantly diminished or abolished 17.

Regarding recombinant viral vector vaccines, viruses other than SARS-CoV, which were capable of host cell infection, were genetically modified to express SARS-CoV components 18. VLPs were non-infectious multi-protein complexes derived from viral proteins, which were self-assembled into structures similar to viruses 19. So far, inactivated SARS virus vaccines, DNA vaccines, and subunit vaccines targeted for SARS S glycoprotein have entered phase I clinical studies 20-22. It is indispensable in vaccine development to confirm that the vaccines defend against viral infection and subsequent diseases. This is generally achieved by introducing infectious viruses to the vaccinated animals and individuals. Challenge experiments in humans were not conducted because of the SARS-CoV; thus, the safety and effectiveness of the vaccines were not tested and confirmed.

**Figure 2:**
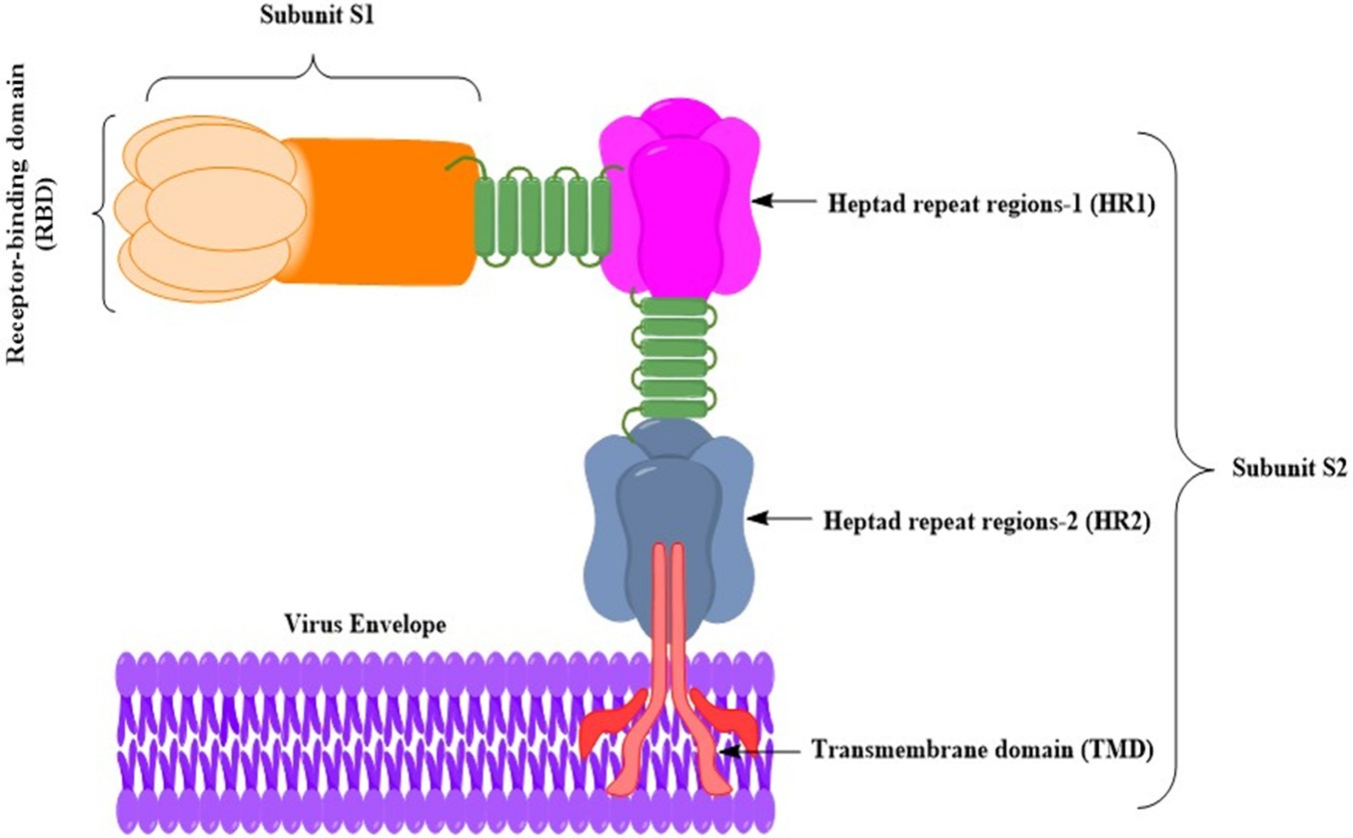
Schematic diagram of the MERS-CoV S glycoprotein anchoring to the virus envelop 27. The MERS-CoV S glycoprotein has been a target for developing MERS vaccines. S glycoprotein gives rise to substantial titers of neutralizing antibodies, and the antigens in subunit vaccines have been often manipulated. The S glycoprotein attaches via the S glycoprotein RBD to the host cell receptor dipeptidyl peptidase 4 (DPP4) 28. It is possible to divide the S glycoprotein into two subunits (S1 and S2). The RBD includes the subunit S1. The S2 subunit contains (HR1 and HR2), which are used by MERS-CoV for membrane fusion and host cell entry. The S glycoprotein is portrayed as a class I fusion protein and forms a trimmer.

Since its appearance in 2012, a variety of vaccines have been developed for MERS-CoV. As is the case with SARS vaccines, most MERS vaccines were predicated on the S glycoprotein 10. Inactivated virus vaccines, live attenuated virus vaccines, recombinant viral vector vaccines, nanoparticle vaccines, DNA vaccines, and subunit vaccines were developed and evaluated mainly in animal models (**Fig. 2**) 23. Whereas modified vaccinia virus Ankara (MVA)-based vaccines and adenovirus-based vaccines have been pending in phase I clinical trials, a DNA-based vaccine has been evaluated 24-26.

## SARS-CoV-2 coronavirus vaccine

**Figure 3:**
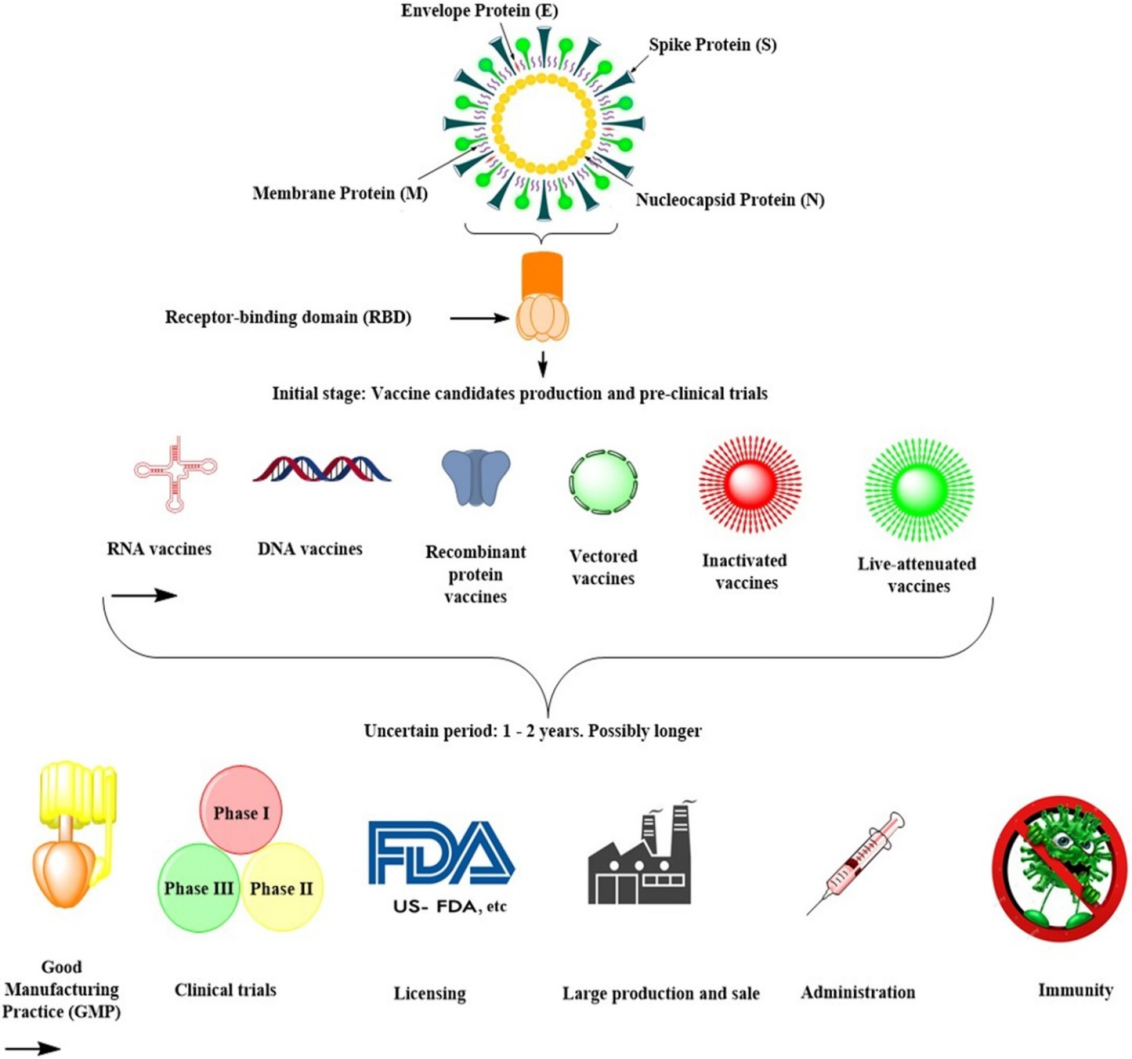
Summary of SARS-CoV-2 vaccine development in future.

Developing human-use vaccines would take several years and possibly several million dollars, especially when using new technologies that have not been exhaustively examined for safety or expanded to mass manufacturing 29. Since there have been no coronavirus vaccines on the market, and there has been no large-scale processing capability for such vaccines as yet (**Table 1**), such processes and technologies would need to be developed. They could be complex, exacting, and time-consuming for the first time (**Fig. 3**). The Coalition has awarded several new sophisticated players in the field funding for Epidemic Preparedness Innovation (CEPI). Most of them are likely to be successful in having made SARS-CoV-2 vaccines 29.

Nevertheless, none of these organizations and entities have an existing pathway to put such a vaccine into late-stage clinical trials, which enable regulatory agencies to approve. At present, they cannot manufacture the number of doses required. An mRNA-based vaccine, in which mRNAs are encapsulated in lipid nanoparticles, expresses a target antigen* in vivo* after the infusion. It was co-developed by Moderna and the National Institutes of Health Vaccine Development Center, which has recently launched a phase III clinical trial (ClinicalTrials.gov: NCT04470427) 29. Curevac has been developing a similar vaccine, which is still in a pre-clinical stage 29. Many vaccines, such as recombinant protein-based subunit vaccines, viral-vector vaccines, DNA vaccines, live attenuated vaccines, and inactivated virus vaccines, are now in the pre-clinical stage (**Fig. 3; Table 1**). Since all of these strategies have both benefits and drawbacks, it is difficult to define which approach would be quicker or more effective (**Table 1**).

Johnson *et al.* and Sanofi have developed vaccine candidates for SARS-CoV-2 30, 31. Johnson *et al.* used an experimental adenovirus-based vector vaccine that has not yet been culminated as an approved vaccine 30. Sanofi's vaccine might be available for use in the human community within months to years because the SARS-CoV-2 vaccine's manufacturing procedure is identical to that used for their licensed Flublok recombinant influenza virus vaccine 32. On April 20, 2020, Qiang Gao *et al.* established a pilot-scale manufacturing procedure of an inactivated SARS-CoV-2 virus vaccine (PiCoVacc) as a refined vaccine candidate 33. They demonstrated that the vaccine provided complete protection to non-human primates by triggering effective humoral immune responses to combat the systemic spread of SARS-CoV-2 33. The study is the world's first publicly reported animal trial of the SARS-CoV-2 vaccine. The PiCoVacc vaccine developed by the team induced the production of SARS-CoV-2 specific neutralizing antibodies in mice, rats, and rhesus monkeys that effectively neutralized 10 representative SARS-CoV-2 strains. Immunizing rhesus monkeys with the PiCoVacc vaccine at two different doses (3 μg or 6 μg per monkey) resulted in a partial protective effect when they were administered with 3 μg of the vaccine and a complete defensive impact when with 6 μg of the vaccine 33.

Moreover, no enhancement of antibody-dependent infection or immunopathological deterioration was observed 33. The PiCoVacc vaccine was evaluated systematically by monitoring clinical signs, blood biochemistry, and histopathological analysis in rhesus monkeys. It was concluded that the PiCoVacc vaccine was safe and well-tolerated 33. The experimental results indicate that the PiCoVacc vaccine is likely to have a spectral neutralization effect on the global outbreak of SARS-CoV-2 33. Preliminary results of EJ Anderson *et al*. found that adverse effects correlated with the mRNA-1273 vaccine were predominantly mild or moderate in older adults in a small number of patients. This group is especially at risk of disease and death from COVID-19 34. The 100 μg dose produced stronger binding-and neutralizing-antibody titers than the 25 μg dose, results that endorse the continued assessment of the 100 μg dose level and the two-dose regimen in a broad. More diverse, phase 3 population-based trials have been developed to evaluate the safety and effectiveness of the mRNA-1273 vaccine and determine its defense level against COVID-19 34. This data should support the rapid clinical development of a human SARS-CoV-2 vaccine.

Kumar *et al*. noted that one problem appeared during the COVID-19 pandemic, where various countries are in a rush to make a SARS-CoV-2 vaccine, particularly the United States, Russia, and China. At the same time, countries such as the United States and Russia provide for their citizens a large and adequate number of COVID-19 vaccine doses and prioritize their market instead of making them accessible to many other countries 35. This is generally referred to as '*vaccine nationalism*.' This could be achieved by a vaccine manufacturer and the government by pre-purchase deals—the WHO has alerted against vaccine nationalism, as it would support the virus rather than help humanity. As a similar event was reported during the H1N1 flu pandemic in 2009, this is not a new problem 35. At the time, Australia was the pioneer among vaccine manufacturers for H1N1 flu and the government prohibited exports; however at the same time, with some pharmaceutical giants, the rich countries went for pre-purchase deals. The United States government has indicated an interest in securing 600,000 doses in the event of COVID-19 35. Regrettably, while vaccine nationalism is against global public health values, there is still no legislation banning the pre-purchase of pandemics such as COVID-19.

## The challenges of creating SARS-CoV-2 vaccines

The empirical-based vaccine companies have achieved important improvements to human health in the past decade 36. Nevertheless, in terms of current immunology and molecular microbiology, vaccine research is still immature, leading to the requirement of a more extended period to produce a new vaccine 36. Increased health issues, highly sophisticated production procedures, and related research criteria have to be considered scrupulously and meticulously when developing new vaccines. In order to address overlapping medical, technological, regulatory, and public safety requirements, a new collection of rules and guidelines would be required if a SARS-CoV-2 vaccine is developed on a fast-track basis for possible clinical use 36.

A correlation between immune responses and protective effects has been gradually questioned for nominee vaccines in recent years 37. The preparation of structure-guided antigens is very popular. At the same time, advances in vaccine development are very far from being an established research field. After nearly four decades of studies, the cessation of HVTN702 announced recently informed us again of a significant discrepancy between research and the development of human immunodeficiency virus (HIV) vaccines 36. Can the protective function of antibodies be the main immune responses to SARS-CoV-2 for specific vaccine programs? The obstacle-free animal models would be highly useful for choosing the candidates for clinical trials, despite an inherent difficulty in coordinating feasibility trials efficiently.

Current vaccine development requires a spectrum of requisite skill sets. Over the last two decades, a broad range of innovations has arisen 38. Considering complex vaccine specifications against quickly spreading new viral infections, vaccine technologies with prior human research experience would have considerable benefits, especially health concerns. Additionally, it is worthy of note that the inventor could rapidly push his/her vaccine development into a scale-up Good Manufacturing Practice (GMP) output with theoretically 10-million doses. Those with an existing facility and manufacturing expertise would be in a far more favorable position 36.

The challenge confronting regulatory agencies for the rapid development of SARS-CoV-2 vaccines is close to that of vaccine producers. High-level considerations would be paid to the health assessment of candidate vaccines against SARS-CoV-2. Immunopathogenesis of the virus plays a crucial role in the SARS-CoV-2 infection, ensuring that vaccination against such a virus would not induce the same forms of adverse immune reactions. This would affect the form of vaccines and the expected immunogens to be chosen. Would a competent manufacturing cycle be adequate for nominee vaccines to proceed, or will it be validated? Will the production and regulatory expertise acquired in other countries be used to evaluate an application for a vaccine in the current country? Is it necessary to consider cell banks or other intermediate goods through national borders? Can the political or economic concerns become obstacles for worldwide efforts to resolve the immediate need for SARS-CoV-2 vaccines? Eventually, the preparation will now begin to allow the globe to have equitable access to effective SARS-CoV-2 vaccines, if the worldwide needs occur. The following concerns have to be resolved: vaccine ownership, unparalleled development financing, the pricing and supply network, and the coordinated delivery of such vaccines to achieve complete pandemic control.

## Vaccination strategies based on the pathogenesis of coronavirus in the host

A better understanding of SARS-CoV-2 pathogenesis, protective immunity, and natural immunity duration will assist in the development of SARS-CoV-2 vaccines 39. An overview of SARS-CoV-2 pathogenesis, including the infected target organs and the transmission route to certain organs, can help develop vaccines to interfere with viral propagation and avoid target organ infections 39. Whether SARS-CoV-2 targets the lungs to induce pneumonia through viremia or after upper respiratory infections are a significant consideration. Live replicating vector vaccines and attenuated virus vaccines for influenza infections efficiently trigger local mucosal immunity, resulting in the shield of the upper and lower respiratory tracts and the limitation of nasal shedding 39. Even though live attenuated influenza virus vaccines primarily induce local immunoglobulin A (IgA) antibodies but less systemic IgG antibodies, they protect influenza infections 40.

Conversely, if the lungs are not the primary sites of SARS-CoV-2 infections, intramuscular parenteral (IM) vaccinations that mostly induce serum virus-neutralizing (VN) antibodies to prevent viremia and are also transuded into the lungs would prevent SARS-CoV-2 infections 39. It might be similar to the mechanism underlying eliminating human respiratory infections by inactivated influenza vaccines via IM. Alternatively, a parenteral vaccine itself, including an S subunit or the receptor-binding domain (RBD) of the S subunit, could be useful as an annual booster vaccination in people who have convalesced from SARS-CoV-2 infections, again, like seasonal influenza 39. This would improve memory B- and T-cell responses and tolerance, and prevent them from being re-infected with the viruses. For certain SARS-CoV-2 cases, diarrhea and fecal shedding have been recorded, and thus oronasal vaccinations could be more appropriate in this scenario 41. Three populations should, therefore, be considered in SARS-CoV-2 vaccinations: vulnerable individuals with no immunity; convalescent individuals with varying degrees of immunity like subclinically affected people; and those with pre-existing immunity to SARS and MERS 39. Consequently, the immunogenicity, protective ability, or negative impacts of nominee vaccines can differ across such categories. Evaluating pre-existing immunity levels would be critical to confirm the vaccines' effectiveness and protection in each community and the various age categories within each community, particularly in the elderly with the highest mortality rates.

## Major strategies for developing SARS-CoV-2 vaccines

### Whole Virus Vaccines

Live-attenuated virus vaccines and inactivated virus vaccines are prepared based on conventional production procedures. According to company reports, Johnson & Johnson is one of the multi-national pharmaceuticals that set out to develop SARS-CoV-2 vaccines 42; Based on their Ebola vaccine design, they employed Janssen's AdVac^®^ adenoviral vector and its PER.C6^®^ cell line technologies for manufacturing a vaccine 42, 43. Additionally, researchers at Hong Kong University have produced a live influenza vaccine co-expressing SARS-CoV-2 proteins 44. Codagenix has developed a technique for “codon deoptimization” to attenuate viruses and develop vaccination methods for SARS-CoV-2 45. A critical benefit of whole virus vaccines is their immunogenicity to activate toll-like receptors (TLRs), like TLR3, TLR7/8, and TLR9, which are expressed on innate immune cells. Nonetheless, it is indispensable for live viruses to be examined for safety profiles and protective effects. It is, especially, a prerequisite to determine whether or not antibody-dependent enhancement occurs after vaccination with live or killed SARS-CoV-2 virus vaccines 46.

### Subunit Vaccines

In SARS-CoV vaccines and SARS-CoV-2 vaccines, the most palpable targets are S proteins in the development of subunit vaccines, which would lead to inhibition of the binding of the viruses to the host angiotensin-converting enzyme 2 (ACE2) 46. The University of Queensland is now synthesizing viral surface proteins under support from the Coalition for Epidemic Preparedness Innovations (CEPI), which activates the host immune system more effectively. In addition, Novavax created and manufactured immunogenic, virus-like nanoparticles encompassing recombinant S-proteins 47. However, Clover Biopharmaceuticals is developing a subunit vaccine composed of a trimerized SARS-CoV-2 S-proteins utilizing its proprietary Trimer-Tag® technology 48. In contrast, full-length S-proteins might induce enhanced infectiveness and eosinophilic infiltration following the challenge by SARS-CoV-2. Consequently, a group headed by the Texas Children's Hospital Center for Vaccine Production at Baylor College of Medicine (such as the University of Texas Medical Branch and the New York Blood Center) produced and validated a subunit vaccine consisting of the receptor-binding domain (RBD) of SARS-CoV S-protein 46, 49, 50. The SARS-CoV RBD vaccine adsorbed on alum induces elevated protective immunity levels to the threat of homologous viruses. The RBD-based vaccine has an advantage in its potential to reduce host immunopotentiation 46. SARS-CoV and SARS-CoV-2 RBDs have an amino acid identity of more than 80% and bind to the same ACE2 receptor, indicating that the strategy for developing vaccines for SARS-CoV-2 could be utilized for the development of SARS-CoV vaccines 51.

### Nucleic Acid Vaccines

Several pharmaceutical companies have developed SARS-CoV-2 nucleic acid vaccines. For example, Inovio Pharmaceuticals designed a DNA vaccine, although other companies have been pursuing RNA vaccine strategies, including Moderna Therapeutics and Curevac 51. In 1993, it was demonstrated that DNA vaccines induced protective immunity against influenza in mice models. However, these nonclinical studies have not yet been translated into clinical studies in humans for decades 51. More recently, much improvement has been made in nucleic acid vaccines' formulations, increasing safety. Whereas nucleic acid vaccines are currently used for only animals, it is getting more likely that nucleic acid vaccines could be applied to humans.

## Selection of antigens

### Whole-cell antigen (WCA)

Includes all the virus elements, including proteins, lipids, polysaccharides, nucleic acids, and other structural and non-structural components. Killed and live-attenuated virus vaccines are typical WCA vaccines 52, 53. Considering WCA's diverse formulations, it is important to carefully monitor the quality assurance and the performance evaluation. To date, many institutions have effectively identified SARS-CoV-2 virus strains and have begun creating killed or live-attenuated WCA vaccines. It is essential to select strains with low pathogenicity or no pathogenicity, in which stringent screening of vaccine candidates is pivotal 54.

### Spike protein (S protein)

S protein is probably the most promising source of antigens for developing SARS-CoV-2 vaccines. Firstly, it is a virus surface protein and is explicitly identified by the host immune system 55. Secondly, it mediates the interaction between the host cells and viruses via ACE2 as an entry receptor, thereby inducing pathogenicity 55, 56. In addition, homologue proteins have also been used for the development of SARS-CoV and MERS-CoV vaccines, which are effective in nonclinical studies 16, 57-60. The SARS-CoV-2 S protein monomer comprises 1273 amino acids, with a molecular weight of about 140 kDa. Self-association assembles the S protein spontaneously into a homo-trimer, similar to other first-generation membrane fusion proteins (viral fusion protein Class I). The S protein is comprised of two subunits (S1 and S2). The subunit S1 is further divided into two domains, the N-terminal domain (NTD) and the C-terminal domain (CTD), in which the RBD is encoded within CTD. The S2 subunit comprises the essential elements necessary for membrane fusion, including an internal membrane fusion peptide (FP), two 7-peptide repeats (HR), a membrane-proximal external region (MPER), and a transmembrane domain (TM) 61. The structure of the SARS-CoV-2 S trimer in pre-fusion confirmation and that of the RBD domain bound to ACE2 have recently been solved, offering useful information on S protein-based development vaccines 55, 56. To date, the full-length S-protein, the RBD domain, the subunit S1, NTD, and FP have been developed as possible vaccines.

### Nucleocapsid protein (N protein)

N protein is highly conserved among CoVs, with a molecular weight of around 50 kDa. This protein is involved in nucleocapsid production, budding virus signal transduction, RNA replication, and mRNA transcription 62. N protein has been documented to be strongly antigenic, inducing antibodies to this antigen in 89% of patients who suffered from SARS 63. In vaccinated C57BL/6 mice, DNA vaccine encoding SARS-CoV N protein induced a high level of N protein-specific humoral and cellular immune responses and reduced viruses' titer markedly 64. In addition, N protein vaccines of avian contagious bronchitis virus induced the activation of cytotoxic T lymphocytes (CTLs) and resulted in a decrease in clinical signs and lung clearing, indicating that N protein-mediated cellular responses are important in the defense against virus infections 65, 66.

On the other hand, other studies revealed that the N protein immunization did not lead substantially to the production of neutralizing antibodies and did not offer protection against infections in hamsters 67. These findings indicate that the efficacy of N protein-based SARS-CoV-2 vaccines is not guaranteed. However, owing to its strong immunogenicity, N protein itself can be used as a marker in diagnostic assays.

### Membrane protein (M protein)

M protein is a transmembrane glycoprotein with a molecular weight of approximately 25 kDa and is involved in viral replication. This protein exists abundantly on the surface of SARS-CoV 68. Immunization of the full-length M protein-induced neutralizing antibodies in patients with SARS 69. Immunogenic and structural analyses demonstrated that a T-cell epitope cluster capable of triggering a robust cellular immune response exists in the M protein 70. M protein is also highly conserved in many virus species to be used as a target antigen for SARS-CoV-2 vaccine development 68.

### Envelope protein (E protein)

E protein consists of 76-109 amino acids and has ion-conduction properties. It has been shown that E protein could be an inducer of inflammasomes, leading to the production of IL-1β and, ultimately, strong inflammatory responses. It is suspected that E protein is responsible for cytokine storm in patients with SARS-CoV 71. It might be, therefore, difficult to control immune responses after immunization with E protein-based vaccines. In this context, E protein is not ideal as an immunogen in vaccines' development, in contrast to S, N, and M proteins.

## Safety

There are very few reports on the safety of vaccines against SARS and MERS. The introduction of the antibody-dependent enhancement (ADE) and other harmful effects resulting from vaccination or natural re-exposure is problematic. ADE is a mechanism that takes place when non-neutralizing antibodies facilitate viral entry to host cells, thereby potentiating virus infectivity 72. ADE has been reported in cats that had been injected with CoV vaccines 73. In SARS animal models, the incidence of ADE cannot be ruled out. In fact, it was reported that several SARS S glycoprotein-based vaccines and inactivated whole virus vaccines caused immunopathologic changes in the lungs and hepatitis in certain animal models 74-77. Additionally, it has been demonstrated that MERS-CoV vaccination causes pulmonary invasion during mice challenge by utilizing an inactivated MERS-CoV vaccine 78. It is worthy of note that animals with SARS-CoV vaccination are not safe (and vice versa) against MERS-CoV infections, leading to harmful consequences following secondary infections 79, 80. Many experiments on the passive transmission of an antibody in mice and research with non-human primates have not found proof of ADE or pathological impacts 81-87.

It was shown that ADE could be avoided by using the truncated form S glycoprotein. The hypothesis is that the RBD or S1 subunit of S glycoprotein is responsible for inducing neutralizing antibodies, and the S2 subunit induces non-neutralizing antibodies causing ADE. Whereas no integrative data support this hypothesis, RBD or S1-based vaccines induced high titers of neutralizing antibodies and had a certain degree of defense in small animals and non-human primates 60, 88-90. Adjuvants are materials that trigger and alter vaccine immunogenicity and protective effects 91. It was demonstrated that a chemical adjuvant-based vaccine did not induce lung immunopathology even after the SARS challenge 92. It is possible that the adjuvant helped to prevent uncontrollable Th2-polarized reactions, where the adjuvant not only improved the effectiveness but also reduced harmful effects related to vaccination.

## Prospects

Little is understood about SARS-CoV-2 etiology, epidemiology, functional origin, pathogenic process, pathological immune responses, and so on. In addition, the host cellular and humoral immune responses to SARS-CoV-2, which are important for the development of vaccines, remain unknown. These issues are to be tackled in the immediate future through fundamental studies for vaccines' effective development. Many countries and R&D institutions have declared their plans for SARS-CoV-2 vaccine development. The preparation of vaccine candidates *per se* is not a formidable task, because the procedure for producing vaccine candidates for SARS-CoV-2 is essentially the same as that for SARS-CoV.

On the contrary, it is extremely difficult to examine many issues, including safety, protective effects, and a consistent vaccine administration level. In general, the safety, immunogenicity, and effectiveness of the vaccine will be tested across three phases of clinical trials. Usually, it requires more than 10 years to launch new vaccines, and more than 90% of the candidates fail to be filed by the regulatory authority. Over the last three decades, a record of about 3,000 vaccine formulations have been applied to the review of the U.S. Food and Drug Administration (USFDA), and less than 20 vaccines have been authorized for sale. For public safety, we have to produce vaccines in compliance with science legislation for development and manufacturing, and stringent laws governing vaccines' selling.

There were 149 mutation sites in 103 sequenced SARS-CoV-2 genomes, and the virus has developed into two different variants, called L and S, in the early stage of COVID-19 in Wuhan. The research also revealed that the two variants displayed significant regional spread and dissemination variations, leading to vaccine design challenges 93. Clinical trials evaluating different medicines are currently underway, hopefully leading to discovering a new medication to combat SARS-CoV-2-related diseases. In addition, the accelerated production and delivery of vaccines are an effective means for terminating the global SARS-CoV-2 pandemic. While vaccines' development is slower than the spread of the pandemic, it would still be essential and required. Firstly, the pandemic continues to expand worldwide, and more and more reported cases are being found, and the inflection point has not been achieved. Secondly, infections with SARS-CoV-2 will become a flu-like seasonal illness, and long coexist with humans 94.

It must be remembered that SARS-CoV-2 has been reported in no more than 6 months, and subsequent studies on pathogenic characteristics and mechanisms of SARS-CoV-2 have only started. Therefore, since evidence and knowledge so far gathered are quite limited and inconclusive, they must be compiled continuously and recorded.

## Conclusion

Experience in vaccine development for CoV strains such as SARS-CoV and MERS-CoV guides the development of SARS-CoV-2 vaccines. ADE and other harmful effects commonly identified with SARS and MERS vaccine candidates should be carefully examined for the safety validation of SARS-CoV-2 vaccine candidates. Although such characteristics were only seen in certain animal models and vaccine regimens, SARS-CoV-2 vaccine candidates' potential remains to be regarded. Furthermore, as previously recognized in the SARS-CoV and MERS-CoV vaccine development, the risk of the short-term immunogenicity resulting from neutralizing antibodies following natural infection should be cautiously considered in the development of SARS-CoV-2 vaccines.

There ought to be an awareness of the possibility of leveraging T-cell responses in CoV vaccination (alongside B-cell responses). Such responses in animal models were proved to be durable and defensive. Moreover, there is evidence of the longevity of humans, importantly the long term. Strategies such as novel adjuvants, S-glycoprotein tailoring, administration routes, and the usage of as yet undiscovered vaccine technologies to improve immunogenicity and avoid any adverse consequences should also be addressed. It should be stated that the use of the CoV N protein for vaccination may have many advantages. As described above, long-term protective immunity can be expected by utilizing this antigen. It should be mentioned that the proof of short-term immunogenicity and safety in humans does not necessarily imply that the vaccine administration is feasible. Vaccinations prove this argument for certain diseases already eradicated that lack spontaneously acquired immunity. The development of SARS-CoV-2 vaccines will also improve our knowledge and experience in reducing globally relevant pathogenic microorganisms.

The development of vaccines for SARS-CoV-2 was too late to control the first wave of COVID-19. Nevertheless, they may be valuable, when subsequent waves appear later or in a post-pandemic situation where SARS-CoV-2 spreads as a seasonal virus. Furthermore, lessons gained from managing this outbreak should enable us to be more equipped for the future. Viruses are likely to keep coming.

## Figures and Tables

**Table 1:** A summary of SARS-CoV-2 vaccine development platforms

Platforms	Benefits	Drawbacks
Inactivated vaccines	An easy procedure used by many approved human vaccines, current facilities may be used, SARS-CoV adjuvants have been evaluated in humans and may be used to improve immunogenicity.	Vast quantities of the contagious virus must be treated. The integrity of the antigens and/or epitopes must be verified.
Live attenuated vaccines	Existing technology can be utilized with simple procedures required by many approved medical vaccines.	Because of its full genome size, it requires time to build infectious clones for attenuated coronavirus vaccine seeds. There would have to be thorough monitoring of safety.
Viral vector-based vaccines	There is no need to diagnose a contagious virus, outstanding preclinical, and clinical evidence for other new infections, such as MERS-CoV.	Vector immunity may have a detrimental impact on the efficacy of the vaccine (based mostly on vector selected).
Subunit (recombinant protein) vaccines	There is no need to treat an infectious virus; adjuvants may be utilized to enhance immunogenicity.	The capacity to produce recombinant proteins for global use could be limited. The integrity of the antigens and/or epitopes must be tested. Yields have to be high enough.
DNA vaccines	There is no need to manage the contagious virus, fast scaling up, low cost of processing, high heat stable, SARS-CoV testing in humans, quick development feasible.	To achieve strong immunogenicity, the vaccine requires different distribution systems.
RNA vaccines	There is no need to treat a contagious virus; vaccinations are usually immunogenic and likely fast development.	Reactogenicity-related safety concerns were identified.
